# Guidelines for Biomarker of Food Intake Reviews (BFIRev): how to conduct an extensive literature search for biomarker of food intake discovery

**DOI:** 10.1186/s12263-018-0592-8

**Published:** 2018-02-20

**Authors:** Giulia Praticò, Qian Gao, Augustin Scalbert, Guy Vergères, Marjukka Kolehmainen, Claudine Manach, Lorraine Brennan, Sri Harsha Pedapati, Lydia A. Afman, David S. Wishart, Rosa Vázquez-Fresno, Cristina Andres-Lacueva, Mar Garcia-Aloy, Hans Verhagen, Edith J. M. Feskens, Lars O. Dragsted

**Affiliations:** 1 Department of Nutrition, Exercise and Sports, University of Copenhagen, Copenhagen, Denmark; 2 Nutrition and Metabolism Section, Biomarkers Group, International Agency for Research on Cancer (IARC), Lyon, France; 3 Agroscope, Federal Office of Agriculture, Berne, Switzerland; 4 University of Eastern Finland, Kuopio, Finland; 5 INRA, Human Nutrition Unit, Université Clermont Auvergne, F63000 Clermont-Ferrand, France; 6 UCD, Institute of Food and Health, UCD School of Agriculture and Food Science, University College Dublin, Dublin, Ireland; 7 Division of Human Nutrition, Wageningen UR, Wageningen, The Netherlands; 8 Department of Biological Sciences, University of Alberta, Edmonton, Canada; 9 Biomarkers and Nutrimetabolomics Laboratory, Department of Nutrition, Food Sciences and Gastronomy, University of Barcelona, Barcelona, Spain; 10 CIBER de Fragilidad y Envejecimiento Saludable (CIBERFES), Instituto de Salud Carlos III, Barcelona, Spain; 11 European Food Safety Authority (EFSA), Parma, Italy; 12 University of Ulster, Coleraine, Northern Ireland UK

**Keywords:** Biomarkers, Food exposure markers, Metabolomics, Systematic review, Literature search methodology

## Abstract

Identification of new biomarkers of food and nutrient intake has developed fast over the past two decades and could potentially provide important new tools for compliance monitoring and dietary intake assessment in nutrition and health science. In recent years, metabolomics has played an important role in identifying a large number of putative biomarkers of food intake (BFIs). However, the large body of scientific literature on potential BFIs outside the metabolomics area should also be taken into account. In particular, we believe that extensive literature reviews should be conducted and that the quality of all suggested biomarkers should be systematically evaluated. In order to cover the literature on BFIs in the most appropriate and consistent manner, there is a need for appropriate guidelines on this topic. These guidelines should build upon guidelines in related areas of science while targeting the special needs of biomarker methodology. This document provides a guideline for conducting an extensive literature search on BFIs, which will provide the basis to systematically validate BFIs. This procedure will help to prioritize future work on the identification of new potential biomarkers and on validating these as well as other biomarker candidates, thereby providing better tools for future studies in nutrition and health.

## Background

The importance of diet for improving health and preventing chronic disease is widely recognized. Indeed, one of the main goals of modern nutritional science is to understand the nature of healthy diets in order to bring “healthy nutrition for all” [1]. The measurement of dietary exposure in interventional as well as observational studies is of crucial importance for the discovery of unbiased associations between food intake and health. By far, the most commonly applied tools for estimating dietary exposure are based on self-reporting, such as food frequency questionnaires (FFQ) for the assessment of regular consumption of usual foods and food diaries (FD) or 24-h recalls (R24h) for a more detailed assessment of short-term food intake. However, such measurements often contain systematic and random errors that are inherent to the method used for data collection [2, 3]. The use of biomarkers of food intake (BFIs)^1^, measured in biological samples, may provide a more objective estimate of actual intake, representing a promising complement to the current self-reporting tools [4, 5]. In this context, metabolomics has opened new opportunities for BFI discovery and new putative biomarkers are frequently identified by metabolic profiling of body fluids following the intake of various foods, meals, or diets. Putative BFIs is a term used here for compounds associated with food intakes based on a single explorative study or which has been proposed loosely based on knowledge of food composition and human metabolism. Such markers need further confirmation to support their potential as BFIs before being proposed as candidate BFIs. The candidate BFIs are identified among the putative BFIs by a further selection process, e.g., by confirmation in more human studies, preferably with a different design and/or populations, or by removing implausible entries based on the data collected from the literature [6]. However, well-accepted markers of food intake exist only for a very limited number of foods, and there is a growing interest and an urgent need to discover and evaluate new BFIs, as well as to re-evaluate those suggested in publications outside the metabolomics area. Therefore, experimental studies to identify new BFIs should be complemented by extensive review of the literature on potential pre-existing BFIs. This will not only improve marker identification in metabolomics but also expand the list of compounds for validation as potential BFIs.

The topic of BFIs has been characterized by a continuous increase in the number of publications over the last 20 years.

Several research groups have summarized the most significant findings regarding BFI discovery via untargeted metabolomics in a number of recent reviews [4, 7, 8]. However, a systematic collection and evaluation of the literature available on putative BFIs for specific foods and/or food groups has never been carried out. A systematic approach to the identification of putative and candidate BFIs should follow a rigorous methodology inspired by existing guidance on health and nutrition [9–11]. However, since exposure biomarker analysis and health assessment are quite different fields, a guideline on BFI reviews will, to some extent, include a different set of steps and procedures.

**Fig. 1:**
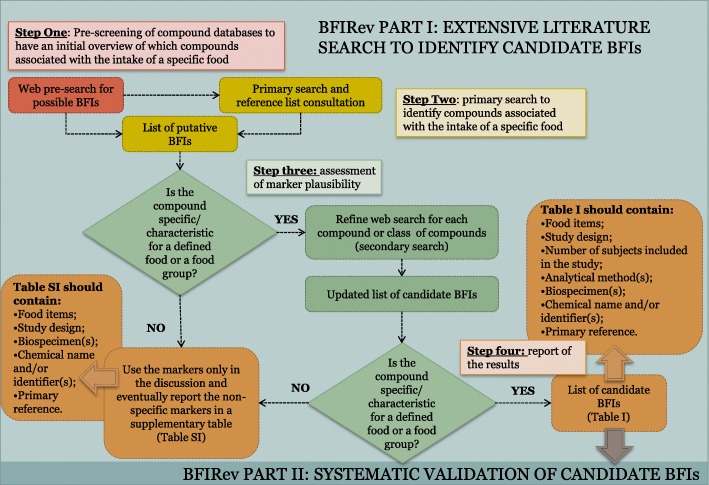
Scheme of the currently suggested BFIRev research methodology. The “Guidelines for Biomarker of Food Intake Reviews (BFIRev)” 4-step procedure is designed for listing candidate food or food group biomarkers (Table I) while also retrieving important information for biomarker validation, whenever it is available. BFIRev is shown here as a decision tree listing the most important steps. The questions in the diamonds should be assessed by at least two researchers independently

In this paper, we propose a strategy to carry out an extensive literature search to identify putative and candidate BFIs, which represents the first part of a guideline for conducting a systematic BFI review, the BFIRev methodology (Fig. 1). The validation step will be the object of a separate paper and will therefore complete the systematic BFI reviewing process.

## Structure of the guideline for a systematic BFI review

The initial step of the present work involved identifying the most important food groups to be reviewed for relevant BFIs. In order to obtain good coverage of the food intake in different population groups within Europe, a list of nine food groups was initially identified by the FoodBAll partners. This was based on country-specific dietary surveys [12] and groupings commonly used in food frequency questionnaires, such as EPIC [13]. These nine food groups and several of their specific subgroups and food items covered are listed in Table 1.

**Table 1:** Principal food groups that need investigation by the BFIRev procedure

Food group and related foods	Food group and related foods
Non-alcoholic beverages	Nuts and vegetable oils
Coffee	Nuts
Tea	Walnuts
Low-calorie sweetener-containing beverages	Almonds*
Sugar-sweetened beverages	Hazel nuts
Alcoholic beverages	Pistachio
Alcohol as such	Macadamia nuts
Beer	Peanuts*
Cider	Brazil nuts
Dessert wine	Other nuts
Red (and rose) wines	Oils
White wine	Olive oil
Whisky, cognac, gin, and other distillates	Sunflower oil
Food of animal origin	Flaxseed oil
Dairy products	Rapeseed oil
Dairy products in general	Legumes
Dairy fat/butter	Peas
Milk	Soy and misu products
Fermented non-solid dairy products	Lentils
Cheeses	Chickpeas
Casein and whey protein	Beans
Meat	Spices and herbs
Meat in general	Anise
White meat	Basil
Pink meat	Black pepper
Red meat	Caraway
Offal meat	Chili pepper
Processed meat	Cinnamon
Cooked and grilled meat	Clove
Fish and other marine food	Coriander
Fatty fish	Cumin
Lean fish (from the sea or from lakes)	Curcumin (Turmeric)
Crustaceans and mollusks	Dill
Fish Oil	Fennel
Eggs and processed eggs	Fenugreek
Fruit and vegetables	Ginger
Fruit and vegetables in general	Lemongrass
Fruit (in a culinary sense)	Marjoram
Berries (strawberry, blackberry, raspberry, blackcurrant, redcurrant,…)	Nutmeg
Pomes (apple, pear, quince)	Oregano
Grapes	Parsley
Citrus (orange, lemon, lime, grapefruit, pummelo, clementine,…)	Peppermint
Banana	Rosemary
Drupes (peach, apricot, nectarine, plum, cherry)	Saffron
Other tropical fruits (pineapple, mango, papaya, kiwi,…)	Sage
Other fruits (muskmelon, watermelon, persimmon,…)	Spearmint
Vegetables	Tarragon
Cruciferous (cabbage, kale, broccoli, cauliflower, brussels sprouts)	Thyme
Root vegetables (carrot, turnip, parsnip, celeriac, radish,…)	Confectionary
Leafy greens (spinach, lettuce, endive, garden rocket)	Cocoa
Fruit vegetables (eggplant, tomato, bell pepper,)	Chocolate
Gourds (pumpkin, cucumber, squash, zucchini)	Liquorice
Allium vegetables (onion, garlic, shallot, leek, chive, ramsons)	Sugar-based sweets (bonbons)
Other vegetables (asparagus, artichoke, celery stalk,…)	Wine gums
Tubers	Other confectionary
Potato	
Cassava	
Yam	
Sweet potato	
Jerusalem artichoke	
Cereals and wholegrain	
Oat and processed oat products	
Barley and processed barley products	
Wheat and processed wheat products	
Rye and processed rye products	
Other grains and grain products	
Rice	
Sorghum	
Mixed cereal products	
Other cereals and wholegrains	

*Although peanuts are botanically classified as legumes and almonds are botanically drupes, they have both been included in the nuts section due to their nutritional profile

The search methodology was drafted based on the literature describing similar search strategies and sent for commenting by all 11 FoodBAll research groups, participating in this activity. In this case, *Allium* vegetables were selected as an example of a food subgroup. The first version of the search strategy where consensus was achieved was later adopted for searches of BFIs for several other food groups and further modified to the current version.

The structure of the present guidelines for conducting an extensive literature search on putative and candidate BFIs follows that proposed by the European Food Safety Authority (EFSA) for conducting systematic reviews for food and feed safety assessments [10], as well as the “Cochrane handbook for systematic review on interventions” [9], with proper modifications for handling BFIs. The PRISMA statement for the reporting and discussion of the results [11] was also used to develop the BFIRev guideline. However, the series of steps finally proposed here have been adapted for literature search on BFIs. These steps are reported below and will be discussed in more detail throughout:

1. Designing the review for a specific food group,
2. Searching for relevant BFI research papers,
3. Selecting and screening papers for quality and relevance,
4. Selection of candidate BFIs and data collection from the selected records,
5. Assessing the quality of the included papers on candidate BFIs,
6. Evaluating the current overall status of BFIs for the food or food group in question,
7. Presenting the data and results,
8. Interpretation and conclusion.

Our methodology has been designed to obtain the most extensive coverage of relevant studies on the discovery and/or application of BFIs in nutritional studies, with a structured and reproducible strategy. Therefore, it will share the framework of systematic reviews for paper searches, screening, and selections (steps 1–4). Nevertheless, the steps for BFI evaluation and study synthesis (steps 5–8) will differ significantly from guidelines for other types of reviews. Table 2 summarizes the steps for the identification and evaluation of BFIs.

**Table 2:** Typical features of an extensive literature search methodology on BFIs

Extensive literature search on BFIs	
Steps	Characteristics of the step
1. Designing the review for a specific food group	*Objective*: Identify and evaluate existing biomarkers for dietary assessment for a specific food or food group.
2. Searching for relevant BFI research papers	*Eligibility criteria for inclusion or exclusion of studies*: Pre-defined and objectively applied. *Inclusion criteria*: Eligible study designs should include any human study with a well-documented intake of the targeted food. *Exclusion criteria*: Defined case by case by objective criteria.
	*Description of the review method*: Systematically documented.
	*Literature search*: Structured in order to identify the highest number of relevant results, documented and reproducible.
3. Selecting and screening papers for quality and relevance	*Defined procedure, documented results*: Identification of a list of publications containing information and/or applications of possible food biomarkers related to the consumption of a specific food or food group.
4. Selection of candidate BFIs and data collection from the included records	*Procedure*: Identification of possible candidate biomarkers and systematic extraction of information to evaluate the usefulness of each compound as BFIs.
5. Assessing quality of included papers on candidate BFIs	*Methodological quality assessment of included studies*: Evaluation of results in intervention and observational studies. Evaluating risk of bias (false positive identification, missing entries).
6. Evaluating the current overall status of BFIs for the food group in question	*Synthesis*: Systematic synthesis of the information to evaluate the specificity and the presence of other quality information (robustness, kinetic properties, dose-response, etc.) on each candidate BFI. Preparing for systematic validation
7. Presenting data and results	*Reporting of study results*: Reporting of the paper containing candidate biomarkers in structured tables and in the text; non-selected markers are listed in a supplementary list.
8. Interpretation and conclusion	*Overall assessment*: The usefulness of the candidate BFIs and/or suggest possible candidate biomarkers or combinations of markers for further investigation and validation.

### Designing the review for a specific food group

In this step of the review process, the objective, review question, and eligibility criteria for study inclusion or exclusion are discussed.

The *objective* of conducting an extensive literature search on BFIs is to list the existing candidate BFIs for a specific food or food group and to provide available evidence for the subsequent systematic evaluation of the quality of such compounds as BFIs.

The *review question* relates to specific intake biomarkers of foods or food groups. Food groups largely include foods of animal or plant origin but may also comprise other sources, as in the case of table salt and certain supplements. Moreover, they differ in their subdivision related to culinary, technological, biological, or nutritional practices. Preparing an extensive literature search of BFIs for foods within a specific food group should therefore start by drawing up the links from the overall food group selected and then dividing the food group into subgroups, all the way to single foods. Taking vegetables as an example, one must initially decide on how to subdivide the group and whether fruits used as vegetables (e.g. tomato, cucumber, eggplant) should be included into the vegetable group. In the next step, the major subgroups such as *Allium*, cruciferous, apiaceous, green leafy, etc. vegetables should be listed, and finally, for the last step, the single foods within these groups should be considered, e.g., for the *Allium* subgroup species such as onion, garlic, leek, shallot, chives, and ransom (Table 1). It is well known that several further subgroupings (including varieties of each of these) exist, such as various red onions or the Vidalia variety of onion, and the detail of the search would depend on the relevance of discriminating between these in nutritional science. For current dietary instruments, this kind of detail is highly variable and it usually does not include varieties, although these are sometimes included in food composition databases [14, 15]. Decisions on how to subdivide and what detail to include has direct consequence for the search profile, as well as for the BFI evaluation step. Therefore, the strategy for each systematic review should aim to identify (i) general BFIs for the food group, (ii) more specific BFIs for relevant food subgroups, and (iii) highly specific markers for selected foods within each subgroup (when this is possible), as proposed in the list of food groups reported in Table 1.

To achieve this goal, it is necessary to identify the key elements that will determine the search questions of the review. This will help in defining the eligibility criteria, the search strategy during the study selection, and the presentation of the results. In reviews of interventions, these criteria represent a combination of clinical aspects (defined by the acronym PICO). PICO specifies the types of population (Participants), Interventions (and Comparisons), and Outcomes [16]. These criteria can easily be translated and adapted in a BFI review. In particular, for the identification and evaluation of existing biomarkers for dietary assessment, we are dealing with descriptive questions about populations, prevalence, occurrence, and consumption in which the population and the outcome of interest need to be specified [10]. The *population* could be the population at large or any subgroup. As the aim of conducting an extensive literature search on BFIs is to identify and evaluate existing biomarkers for dietary assessment, no limitations need to be made on the population characteristics of the subjects. Even though a biomarker may be valid for a specific population, at this stage, the search should not filter for any specific geographical area and should include both healthy volunteers and patients of all ages. However, whenever a defined subgroup is selected for a biomarker study, it must be determined whether this selection might reasonably affect the generalizability of the BFIs.

The expected *outcome* is the existence of a significant relationship between the intake of a certain food or food group and the presence of a specific food-related compound or group of compounds in body fluids or tissues. Such compounds should represent qualitatively and quantitatively the consumption of that food and be robust markers in real-life situations in that other foods or food groups are not likely to yield the same BFI.

One typical feature of an extensive literature search is the a priori specification of *eligibility criteria* for including or excluding studies in the review. Such criteria are guided by the key points previously introduced.

Eligible study designs should include any human study with a well-documented food/dietary intake. This may include the following categories: (i) intervention studies (randomized controlled trials over a period of time or single meal studies) in which the participants consume known amounts of specific foods and in which biological fluids or tissue samples are collected at one or more time points before and after the trial period and (ii) population-based studies (cross-sectional studies, case-control studies, cohort studies) in which the participants are classified and compared as consumers and non-consumers, high- and low-consumers, or with defined strata with respect to the food or food group. Such studies are typically post hoc with biomarker discovery being their main objective. These studies may include existing BFIs or a subjective dietary instrument (e.g., an FFQ or a food diary) to monitor dietary intake. Two main approaches to discover BFIs should be taken into account: the targeted hypothesis-driven approach, based on previous knowledge of food composition [17, 18], and the data-driven approach, provided typically by untargeted metabolomics studies [4]. In the first case, the selection of the investigated marker(s) would be made a priori, based on previous knowledge of a food-specific constituent. In the second case, the markers are not known a priori and an untargeted metabolomics approach is adopted, thereby allowing for the discovery of novel BFIs, as well as confirmation of previously proposed markers.

In cases where no biomarker studies on a food or food component can be found, there may be studies on food compounds that may be specific for that food or food component. Human studies in which specific compounds originating from such foods are provided to volunteers can be used as supportive data on aspects related to absorption, distribution, metabolism, and excretion of that compound. However, these data cannot be taken as evidence that the compound may be a BFI for the food in question. Moreover, papers on nutritional status biomarkers, e.g., related to vitamins or minerals, or effect markers [19], should not be included during the search process for BFIs, as such markers lack specificity for single foods or food groups. Animal studies could be considered especially when human studies are missing and/or when they provide supportive information on biomarkers identified in humans. Consequently, BFIs observed only in animal studies are not eligible as candidate biomarkers but should be seen as putative biomarkers to investigate further in human studies. Exclusion criteria are usually made on an ad hoc basis because the major source of noise in a literature search may come from unpredictable sources such as the author name (e.g., John Trial) or specific wording not directly related to the particular BFI (e.g., allergens in particular food groups). Regardless, inclusion and exclusion criteria must be listed in the method section.

### Searching for relevant BFI research papers

This section outlines the search strategy, selects the sources of information for the review, and identifies the keywords for the literature search. In a BFI review, as for reviews in health and nutrition, authors should list the information sources used, such as the databases searched, the keywords used for the search, and the time period in which the search was conducted. The listed information must also include details on the targeted food group, subgroups, and foods, as well as inclusion and exclusion criteria for the specific literature search.

#### Outline of search strategy

Biomarker of food intake reviews should start out defining as its topic BFIs for a specific food group, subgroup, or single food. The search for the identification of candidate BFIs should be articulated in four steps (Fig. 1). A preliminary screening (step 1) of the food group components should first be conducted in food composition databases (see the “Information sources” subsection) in order to determine which specific compounds may be associated with the intake of the targeted foods or food group. Such a pre-screening step provides a preliminary overview of the compounds known to be present in the targeted food/food group and may help in the following steps of the screening process. Nevertheless, this does not limit the investigation to the food compounds identified in the search or to their known metabolites, since some relevant food compounds and metabolites may not yet have been included in the databases. Following this initial screening, the primary search (step 2) should make use of the resources mentioned in the following section to obtain a list of putative BFIs. This list should be sorted, based on the authors’ knowledge of (a) candidate biomarkers and (b) other compounds, i.e., those known by the authors to be present in many different foods. The division into these two groups of compounds relies heavily on the experience of the researcher and must therefore be cross-validated by an independent expert to avoid further work on implausible markers, such as widespread or even ubiquitous compounds, including most nutrients. However, this is not always straightforward; in the case of *Allium* vegetables, for example, onion is a good source of quercetin and its metabolites are abundant in urine after intake, but quercetin is also well known to be found in many other food items (see also step 3 below). This raises a flag that quercetin metabolites may not be sufficiently specific to be included. In cases of doubt, the marker should be placed initially into the candidate biomarker group. Once the candidate biomarkers have been identified, a second literature search (step 3) should be performed to confirm whether each listed metabolite can be classified as a unique or characteristic marker for the particular food/food group or can also be related to the intake of other foods. This secondary search is also used to obtain additional information (e.g., dose response, ADME (absorption, distribution, metabolism, and excretion) information, and analytical methodology) to evaluate the usefulness of each compound as BFIs (biomarker validation step). In the *Allium* example, quercetin-3,4′-*O*-diglucoside was found by this additional search to be quite specific for onion [20], but its metabolism leads to the presence in urine and plasma of common quercetin metabolites, found after the intake of all plant foods containing quercetin derivatives [21]. Therefore, quercetin may be omitted from the list and retained only if it can be argued that it would form a necessary part of a multi-marker approach, where several biomarkers together provide sufficient specificity for onion. A compound can also be considered unspecific if its endogenous presence in the body is high, making it difficult to discriminate whether the compound is observed as a response to food intake or not. Compound databases, as reported in the “Information sources” subsection, should be used for a first evaluation of potential marker specificity, when the compound of interest is a food compound or one of its expected metabolites. Additionally, the Human Metabolome Database (HMDB) [22] can be used to retrieve information about endogenous metabolites, such as their presence in body fluids and the possible metabolic pathways that lead to the formation of such compounds. As a result of this investigation, a compound should be considered a candidate BFI if it meets one or more of the following criteria: (i) the marker has high specificity for the targeted food or food group, such as arsenobetaine for fish [23] or of alkylresorcinols for wholegrains [24]; long-chain fatty acids might be another example for fish but they are also present in food supplements so they would qualify better for a fish intake biomarker pattern; (ii) the compound is highly characteristic of the food investigated, e.g., markers that are very high in the targeted food compared to others, such as chlorogenic acid for coffee [20]; and (iii) the marker is not fully specific but could be used in a multi-marker approach (e.g., tartaric acid is present in grapes but combined with ethyl-glucuronide may provide a good estimation of wine intake [25]). Clearly, what will constitute a specific biomarker will depend on the population in which the BFI will be applied and later validation steps will include this aspect. However, if none of the three aforementioned criteria are met, the compound should be moved to the list of not plausible markers. The list of candidate biomarkers should be reported in a table (step 4), summarizing the main information relative to the selected studies (see the “Presenting data and results” section).

#### Information sources

The main source of information for the primary search should originate from original research articles searched electronically in relevant databases. In order to get the most comprehensive overview of the scientific papers available, an optimal search strategy should preferably include three databases, including PubMed [26], ISI Web of Science [27], and Scopus [28], as we have observed that the redundancy of the information on BFIs between these databases is quite low. If not all the three databases are available to all research groups, the search could be reduced to two databases if necessary, or another relevant database may be selected. Additional databases, which could be consulted, include Scifinder [29] and Google Scholar [30]. A second source of documents may come from the examination of the reference lists in the relevant articles retrieved. Such an approach may be particularly useful to retrieve older research papers that may not be available through online sources. Relevant reviews and books should be also consulted to manually search additional original literature. For the preliminary screening and the secondary search, the use of compound databases, such as HMDB [22], Exposome-Explorer [31], Phenol-Explorer [20], PhytoHub [32], the Dictionary of Food Compounds [33], and FooDB [34], should be included in the search strategy. Such databases contain information about metabolites detected and quantified in body fluids or in specific foods. Therefore, they could be used to assess the specificity of a certain candidate BFI (see step 3 in the previous section) or to propose new putative markers based on the knowledge of the food compounds. Peer-reviewed literature should exclusively be used, and literature useful to interpret, support, and draw conclusions about biomarker validation should be included when available. For commonly used biomarkers such as EPA and DHA for fish or fish oil supplement intakes, the number of studies may be extremely high and thus highly redundant. Therefore, their inclusion may be limited to recent reviews and meta-analyses but should not exclude methodological studies (e.g., studies on kinetics, analytical methodology, variability, or other aspects of BFI quality). The full list of relevant papers may then be added as a supplement to document the search.

#### Search keywords

The list of search terms for the primary search should be appropriate in order to capture the relevant literature but selective enough to avoid capturing irrelevant ones. The main search strategy should make use of general keywords to limit the search to BFIs, as well as specific terms for the food or food group under investigation. The search should be reproducible in different databases and make use of the Boolean operators “AND,” “OR,” and/or “NOT”; however, the names of search fields to use and filters will vary between databases. In the method section of the BFI review paper, a full electronic search strategy should therefore be reported in the format of at least one of the major databases, including any limits used, so that the search may be reproduced [35].

The selected criteria should be as follows:

1. The first research criterion has to filter the literature for the specific food/food group including all the foods from the food group (e.g., *allium OR onion OR garlic OR leek OR chives OR shallots OR ransom)*. If relevant, the scientific Latin names could also be added as keyword (e.g., *Allium cepa OR Allium sativum*, etc.*).*
2. The next criterion should address the function as a potential biomarker and its metabolism (e.g., biomarker* OR marker* OR metabolite* OR biokinetics OR biotransformation OR pharmacokinetics), where “*” designates a wild character for the search engine used. Further terms could be added, according to the specific information that the scientist would like to obtain (e.g., *metabolism OR kinetics*), but such terms may greatly increase the number of irrelevant results.
3. Further specification of the intake mode will help to filter dietary studies from other clinical studies (intake OR meal OR diet OR ingestion OR consumption OR eating OR drink*). Terms such as (drink* OR *food OR beverage)* may be added when appropriate, but they may add considerable noise to the search results.
4. An additional search string will limit the search to human studies: (human* OR men OR women OR patient* OR volunteer* OR participant*), AND (trial OR experiment OR study) as a minimum. The string could be expanded with *(individuals OR subjects)* for the first string and *(intervention OR cohort OR meal)* for the latter. The decision depends on the signal-to-noise ratio introduced based on a pilot search (e.g., performed by limiting the search to the most recent 2 years).
5. A criterion on samples or specific body fluids will also help to focus the search (e.g., *urine OR plasma OR blood OR serum OR excretion* OR *hair* OR *toenail OR faeces OR faecal water*).
6. Animal studies could be considered as they provide complementary information to human studies. Information from animal studies may be the only available option if information from human studies is missing or lack important information on potential biomarkers found in the preliminary search on food constituents. When taking only human studies into consideration, a NOT operator could be used with a string such as *(animal OR rat OR mouse OR mice OR pig OR …)*. It is important to remember that the NOT operator may also remove several important results, for instance where human and animal studies are published together. Manual removal is therefore recommended. Whenever a NOT operator is used, it is advisable that the removed papers are carefully checked (e.g., searched separately for information on human studies).
7. Further criteria may be added based on the specific food or food group. For instance, in the search for seafood intake biomarkers, “food allergy” could be an important source of noise and might be avoided using the NOT operator (“*food allergy*” OR “*food allergies*”).
8. If the food is consumed after processing such as cooking procedures that may affect the structure of the molecules or produce new compounds (e.g., Maillard reaction products), the processing could be taken into account in the search, e.g., AND *(heated OR cured OR smoked OR…)*.

The criteria outlined above should be combined using AND, except when the NOT operator is specified.

The second literature search, aimed at confirming marker specificity, as well as obtaining further useful information for marker validation, should use the (“compound name” OR “compound class”) as the main keyword, together with AND (biomarker* OR marker* OR metabolite* OR biokinetics OR biotransformation OR pharmacokinetic* OR ADME OR bioavailability). Further filters, such as (urine OR plasma OR serum OR blood OR excretion OR faeces OR faecal water) AND (intake OR meal OR diet OR ingestion OR consumption OR eating OR drink* OR administration) AND (human* OR men OR women OR patient* OR volunteer* OR participant* OR subject*), could be added in order to further filter the result in case the search produces too many irrelevant matches.

### Selecting and screening papers for quality and relevance

The search process outlined above may provide a massive number of records that could be largely irrelevant for the purposes of identifying and documenting relevant BFIs. Therefore, the screening procedure guided by the eligibility criteria should be both efficient and comprehensive. Once the list of criteria to define eligible papers has been defined, at least two parallel reviewers should be identified as advised in the “Cochrane Handbook for Systematic Reviews of Interventions” [36]. The reviewers must independently carry out the assessment of the study eligibility and the extraction of data from study reports. This criterion will help in achieving a consensus between the scientists involved in the review which will also reduce the risk of bias in the evaluation of cause and effect. For primary screening and selection of potential candidate BFIs, an evaluation by two different researchers is advisable but not strictly necessary because there is usually less ambiguity in that part of the evaluation. Instead, two or more expert researchers should evaluate the list of candidate biomarkers following the primary search to make sure that it contains markers to be expected based on prior knowledge. Ambiguity at this step can be resolved by additional primary searches to target any potentially missing candidate markers and by screening any available exploratory untargeted metabolomics studies of markers for the food or food group in question.

In the final extraction of information from the selected papers, it is important to include information useful for further validation of the markers. First of all, the compound information should point to a unique compound identified by an authentic standard. In metabolomics, BFIs are often found and even confirmed in additional studies although the biomarker identity cannot be identified by a standard, because standards are not commercially available or possible to synthesize. If included, such markers should be flagged and information about the uncertainty of their true identity should be clearly mentioned. It should also be argued why such a BFI is included. To state this in terms of the Metabolomics Standards Initiative classification, only level 1 markers (identified by an authentic standard) should usually be included, except in special, well-argued cases.

A typical process for selecting and screening papers for inclusion in a review should include the six steps similar to those proposed for other kinds of review [36, 37]:

1. Merge all the search results from different databases using reference management software and remove duplicate records of the same report.
2. Examine titles and then abstracts for relevance to the study question to remove obviously irrelevant records (authors should generally be over-inclusive at this stage).
3. Retrieve full text of the potentially relevant records.
4. Link multiple records of the same study.
5. Examine full text of the records for compliance with eligibility criteria.
6. Make final decisions on inclusion of the paper or report and proceed to data collection.

The selection process should be described, and results should be reported in a manner that provides the number of studies screened, their assessed eligibility, and those that were included in the review. The reasons for exclusions at each step should also be documented [35]. In particular, review authors should include a study flow diagram as recommended by the PRISMA statement [11, 35] to illustrate the results of the search, the screening process, and the selection of studies for inclusion in the review. The flow diagram should present the number of:

- Unique records identified by the searches.
- Records excluded after preliminary screening (e.g., of titles and abstracts).
- Records retrieved in full text.
- Records excluded after assessment of the full text, with brief reasons for exclusion.
- Papers and reports meeting eligibility criteria for the review.
- Studies contributing to the list of candidate BFIs.

The secondary search may also make use of a similar set of steps, but since it depends on single cases, it is not possible to define a general systematic approach, and the previous framework should be only used as an indicative procedure.

### Selection of candidate BFIs and data collection from the included records

The step following the selection of relevant records consists of identifying candidate BFIs for the food or food group in question and a systematic collection of information for the assessment of the usefulness of the selected compounds as candidate BFIs. Besides the analysis of the full text of papers obtained from the primary search as described above, further information, such as marker specificity and pharmacokinetic properties, could be collected from the records obtained from the secondary search, as stated in the outline of the strategy. Papers should be grouped by the class of compounds in order to facilitate the subsequent evaluation of the information. Such an evaluation targets the evidence that the compound(s) can show an increased concentration or excretion after intake of the targeted food or food group. Ideally, the biomarker signal or its concentration in body fluids or tissues should be very low when the food is not ingested for a sufficient period of time, and it should increase only in response to the food intake and return to baseline at an appropriate time point after the intake ends. Possible information to collect includes whether there is a significant correlation between the candidate biomarker level and the intake of a specific food. In order to confirm the plausibility of the marker as a BFI, it is also important to provide information about its specificity by reporting the relation between the marker and the food composition, including the likely metabolic fate of the parent food compound in the human body. Such data should be supported with information about the study, the population, and the analytical method used to detect and quantify the compound(s), the kinetics of the marker(s), and the existence of a dose-response relationship. Details about the information necessary to evaluate the usefulness of each candidate BFI are reported in the section “Evaluating the current overall status of BFIs for the food or food group in question.” In some cases, the candidate marker may already be present at baseline and/or in the control group, as it could be endogenously produced from low-level secondary sources of exposure. For such candidate BFIs, this lack of specificity may be a serious challenge for their validation. Therefore, information on background exposure and the methodologies used to monitor or adjust for them would be crucial. Candidate BFIs composed of two or more less-specific metabolites should be marked as belonging to this category. Likewise, the reason for keeping them should be stated. As an example, caffeine may be kept as a candidate compound for coffee intake biomarker even though it is present also in tea and in multiple soft drinks, confectionaries, and other convenience products.

All this information is used to shortlist candidate BFIs and will be used more extensively in combination with other biological and chemical information to support marker validation.

### Assessing quality of included papers on the candidate BFIs

In a BFI review, each study should undergo a standardized assessment to evaluate to which degree it is susceptible to bias. In healthcare research, common types of bias can occur in many different study designs. They are often classified as selection, performance, detection, attrition, and reporting biases [10]. Because we are interested in assessing whether a compound found in the body fluids may be used as a BFI (i.e., to estimate compliance, recent or average food intake), the evaluation of the full-text papers in a BFI review will be different from that carried out for reviews on health-related studies. As a result, the risk of bias will differ, especially as it relates to decisions based on the knowledge of the reviewer(s) in areas like food chemistry, human study designs, biomarker theory, and biomarker analytics.

The most common bias may be the over-inclusion of candidate biomarkers. While over-inclusion of candidate biomarkers is advisable in the first part of the review process, unwanted bias may also be seen. For instance, over-inclusion bias could arise from a non-cautious interpretation of correlation analyses in observational studies. Over the last few decades, a significant number of studies have used correlations between metabolites quantified in body fluids and frequency of food intake assessed by FFQ or other self-assessment tools [38, 39]. Even though correction for random and fixed factors is applied, such results may lead to an overestimation of the reliability of the compound as an intake marker. This is because correlation may originate from other co-occurring phenomena and cannot be used to infer causality between the consumption of a food and a change in the measured biomarker. Examples include studies showing unspecific increases or decreases in various lyso-phospholipids [40, 41]. Non-specific BFIs may also be detected in intervention studies, where the background diet is highly controlled, thereby decreasing the robustness of the selected putative marker compounds identified. For instance, hippuric acid has been found as a marker that is changing with a large number of different plant-based foods. Therefore, in a study with a single fruit or vegetable, this marker may seem very important but still be largely irrelevant [42–44]. Another cause of misinterpretation of BFIs could be the unclear boundary between BFIs, effect markers, and biomarkers of nutrient intake. As detailed previously [19], the classification of nutrition and health biomarkers depends on the intended use of the biomarker measurement in the study. Therefore, the reviewer should pay particular attention to identify the purpose for which that compound has been used in a certain work. For instance, average improvements in vitamin A status may have been observed in a deficient population after long-term increased intake of carrots, but this does not mean that retinol (vitamin A) is a good biomarker of carrot intake since many other dietary factors would influence changes in the level of such a compound [45, 46].

Confounders may also originate from the study design. In some intervention studies, the food is administered concurrently with other foods within a meal, with or without a control group. In these cases, the source of the marker may not be clearly distinguished if dietary intake is a mix of several foods, and the study cannot be used as such. However, it may be used to support hypotheses based on more direct evidence from other studies. Other confounders may arise from environmental sources; one example is the polycyclic aromatic hydrocarbons, which could originate from the cooking process rather than from the food itself [47]. In this case, detailed information about exposure and background levels should be presented for a proper evaluation of the suitability of these compounds as BFIs.

Detection bias may also occur in case of limitation in the analytical method or in sample preparation. For example, quercetin-4′*-O*-glucoside was once reported in plasma after onion consumption [48], suggesting that this compound could be a potential candidate biomarker for onion intake. However, it was shown that, even though this compound supported absorption of quercetin faster than other quercetin glycosides [49], its presence in plasma was an artifact [21, 50].

To investigate the characteristics of a compound or metabolite as a BFI, the included studies should present a comparison of consumers and non-consumers of the investigated food. Randomized controlled trials (RCTs) with a crossover design represent the most sensitive kinds of studies for the discovery of BFIs. In this design, the comparison between interventions can be made on a within-participants basis. This is because participants act as their own control, providing a better evaluation of the effect of the treatment (that in this case would be the meal or the diet). Most RCTs are not conducted with a primary aim to observe BFIs, and the control group may be selected with a view of other aims. Selecting a proper control diet is not trivial. For a BFI study, the ideal control diet is highly varied and fully balanced in nutrients while avoiding the specific food or food group in question. In practice, this is often quite difficult to do, and in single meal studies, it is often necessary to design a specific control food nutritionally resembling the food under study. The biomarker study may consequently be contrasting between two foods or food groups, and this must be taken into account in the data analysis. Other studies that could be evaluated are RCTs with a parallel design or quasi-experimental studies with a before-and-after design. The latter design is less robust and cannot be used to assess whether the compound could be a good marker of food intake, but it may be useful for getting additional information on the candidate biomarker, such as its kinetics. Moreover, intervention studies in which different doses of the same food are given to volunteers represent the golden standard to evaluate the existence of a dose-response relationship between the food intake and the presence of the marker in body fluids and tissues. This avoids the uncertainties of questionnaires. On the other hand, these kinds of studies present a highly controlled environment; therefore, the markers observed may not be robust and often need to be validated in further studies, where the background diet is not controlled [51]. Cross-sectional studies provide the optimal conditions to assess the robustness of candidate biomarkers because of the highly variable dietary background and variable intake levels. Case-control studies and prospective studies could be also used to indicate the robustness of the candidate BFIs in free-living subjects. However, if sample collection and food intake assessments are not coincident, the association between intake and measurements may be weak, especially for biomarkers of acute intake. In the case of markers averaging long-term intakes (e.g., carotenoids) or for foods that are very regularly consumed, biomarker concentrations can be compared to habitual food intake as assessed with a FFQ. These kinds of studies can result in useful biomarker validation. It has recently been shown that for many markers, three spot urine samples separated by several months may be sufficient to represent the FFQ for the most commonly consumed foods [52]. Other samples may represent other time frames [53].

Candidate biomarkers may also be initially identified in observational studies and subsequently validated in experimental study designs. However, since most observational study designs are prone to misclassification as well as to confounding factors, confirmation in an experimental study is absolutely necessary in the initial validation of such candidate biomarkers.

### Evaluating the current overall status of BFIs for the food or food group in question

The step following the assessment of candidate BFIs for the food or food group in question consists of a systematic collection of the information from the records obtained from the primary and secondary searches to evaluate the usefulness of such compounds as BFIs. Such a collection of information should prepare the reviewers for the systematic validation of BFIs proposed by our team in a separate paper and should therefore follow the same biological and chemical evaluation criteria. Biological information that should be reported includes:

- Marker plausibility (e.g., if the marker is specific for a certain food),
- Dose-response relationship between quantity of food ingested and biomarker response,
- ADME and individual variability,
- Cumulative aspects (e.g., accumulation in tissues),
- Robustness, that is the ability of the biomarker to indicate the intake of a specific food regardless of complex meals or diets, food matrix, and individual and environmental factors, and
- Reliability, which indicates whether a candidate marker has been validated against other already validated methods, such as other already validated BFIs or dietary assessments.

Analytical aspects should include:

- Information on the chemical stability of the compound,
- Details on the method validation, and
- Analytical reproducibility across laboratories.

### Presenting data and results

The study selection process typically leads to a list of publications containing information and/or applications of putative biomarkers related to the consumption of a specific food or food group. As described in the search strategy, a second literature search should be performed to confirm the specificity of each marker, thereby providing the list of candidate biomarkers. The records containing information on candidate BFIs should be reported in a table, as described below (Table I in Fig. 1), while non-specific markers should be only discussed in the text and the reason of their exclusion should be explained. Reports including only non-specific markers may be reported in a supplementary table in order to provide all the information collected during the systematic search (Table SI in Fig. 1). The BFI table should contain the following information:

- Food items that associate with the candidate marker;
- Study design;
- Number of subjects included in the study;
- Analytical method(s) applied to identify or quantify the marker;
- Biospecimen(s) analyzed in the study;
- Chemical name or trivial name and/or identifier(s) of the candidate biomarker compound(s); identifiers may be InChI key(s) for the candidate biomarker compound(s) or another unique identifier (e.g., Pubchem ID, Chemspider ID, …);
- Primary reference in which the compound has been identified or tested.

For the supplementary table reporting the records with non-specific markers, a column reporting the exclusion rationale should also be added. Anyway, for such table information such as the number of subjects, the analytical method may not be required.

### Interpretation and conclusion of the review

This section should include a description of the quantity and quality of the evidence supporting the review question, the interpretation of the results, any potential limitations of the review process, and agreements or disagreements with other research [10]. In the process of carrying out a BFI review, the reviewers should now have a list of compounds which are either specific or non-specific for the food or food group in question, as well as the necessary information to support their validation as BFIs. Both specific and non-specific markers should be discussed in the text, underlining the point(s) of weakness of each non-specific marker. These reasons may include variations in metabolism or the presence after intake of other food groups. Additionally, the strength of the most promising candidate biomarkers (e.g., specificity for a certain food or food group, existence of dose-response relationship, robustness in real life situation, etc.) should also be discussed in the text. Once the candidate biomarkers have been proposed and evaluated for specificity, robustness, and sensitivity, information regarding their ADME (absorption, distribution, metabolism, and excretion) should be used to further evaluate the performance of the marker as a BFI. Other issues to be evaluated include sample collection and preparation, as well as the analytical method, which should be simple and easy to reproduce. For example, collection of biopsies is a highly invasive procedure, and except for special cases, this is commonly avoided in nutrition-related studies.

The resulting list of putative BFIs should represent the best current knowledge and therefore also points to current knowledge gaps. The putative BFIs still need careful validation in order for them to be used in nutrition studies [6]. The list as such also represents a resource for development of analytical procedures for food intake or compliance assessment and for work on feature identification in metabolomics studies on BFIs. Similar procedures should work also for other biomarkers of dietary exposure, i.e., for nutrient intake biomarkers or non-nutrient intake biomarkers, but this would need to be carefully considered in future work.

## Conclusion

Guidelines for conducting a biomarker of food intake review (BFIRev) have been detailed as an 8-step process. Based on the information collected by an extensive literature search for BFIs for a specific food or food group, strengths and weaknesses of each candidate biomarker can be critically evaluated. This prepares for further validation to assess to which extent the candidate biomarker could actually be considered a fully validated BFI.

The BFIRev guidelines help in listing all known candidate BFIs and prepare for further validation steps by compiling the relevant studies and by examining the strengths and weaknesses of these studies for the validation process. Conducting the BFIRev by these guidelines additionally points out knowledge gaps and consequently the specific needs for additional studies and/or additional information necessary to fully validate each BFI.

## Footnotes

1 Please note that this abbreviation differs from the abbreviation, FIB, used in other papers in this special issue.
